# Validation of genomic and transcriptomic models of homologous recombination deficiency in a real-world pan-cancer cohort

**DOI:** 10.1186/s12885-022-09669-z

**Published:** 2022-05-28

**Authors:** Benjamin D. Leibowitz, Bonnie V. Dougherty, Joshua S. K. Bell, Joshuah Kapilivsky, Jackson Michuda, Andrew J. Sedgewick, Wesley A. Munson, Tushar A. Chandra, Jonathan R. Dry, Nike Beaubier, Catherine Igartua, Timothy Taxter

**Affiliations:** Tempus Labs, 600 W Chicago Ave Ste #510, Chicago, IL 60654 United States

**Keywords:** Gene expression profiling, Diagnostic biomarkers, Homologous recombination\

## Abstract

**Background:**

With the introduction of DNA-damaging therapies into standard of care cancer treatment, there is a growing need for predictive diagnostics assessing homologous recombination deficiency (HRD) status across tumor types. Following the strong clinical evidence for the utility of DNA-sequencing-based HRD testing in ovarian cancer, and growing evidence in breast cancer, we present analytical validation of the Tempus HRD-DNA test. We further developed, validated, and explored the Tempus HRD-RNA model, which uses gene expression data from 16,750 RNA-seq samples to predict HRD status from formalin-fixed paraffin-embedded tumor samples across numerous cancer types.

**Methods:**

Genomic and transcriptomic profiling was performed using next-generation sequencing from Tempus xT, Tempus xO, Tempus xE, Tempus RS, and Tempus RS.v2 assays on 48,843 samples. Samples were labeled based on their *BRCA1, BRCA2* and selected Homologous Recombination Repair pathway gene (*CDK12, PALB2, RAD51B, RAD51C, RAD51D*) mutational status to train and validate HRD-DNA, a genome-wide loss-of-heterozygosity biomarker, and HRD-RNA, a logistic regression model trained on gene expression.

**Results:**

In a sample of 2058 breast and 1216 ovarian tumors, BRCA status was predicted by HRD-DNA with F1-scores of 0.98 and 0.96, respectively. Across an independent set of 1363 samples across solid tumor types, the HRD-RNA model was predictive of BRCA status in prostate, pancreatic, and non-small cell lung cancer, with F1-scores of 0.88, 0.69, and 0.62, respectively.

**Conclusions:**

We predict HRD-positive patients across many cancer types and believe both HRD models may generalize to other mechanisms of HRD outside of BRCA loss. HRD-RNA complements DNA-based HRD detection methods, especially for indications with low prevalence of BRCA alterations.

**Supplementary Information:**

The online version contains supplementary material available at 10.1186/s12885-022-09669-z.

## Background

Genomic instability is an enabling characteristic of cancer that is often mediated by deficiency in DNA damage sensing and repair processes [1]. The homologous recombination repair (HRR) pathway, which is responsible for repairing DNA double-strand breaks (DSBs) [2], is frequently dysregulated in cancer, leading to the accumulation of genomic defects and cancer progression [3]. *BRCA1* and *BRCA2* are foundational to the HRR pathway and were initially discovered due to their association with hereditary breast and ovarian cancers. Since their discovery, screening for germline BRCA alterations has become a powerful tool for clinical risk assessment and management [4]. In addition, understanding the role of *BRCA1* and *BRCA2* in HRR has led to the development of targeted therapies, such as poly-ADP ribose polymerase (PARP) inhibitors. This class of drugs exploits synthetic lethality with the base excision repair pathway to directly target the underlying mechanism contributing to tumorigenesis [5, 6].

Genetically or epigenetically driven loss of function in *BRCA1* or *BRCA2* is the canonical driver of the homologous recombination deficiency (HRD) phenotype, which is defined as the inability to repair DSBs with HRR [7]. Nevertheless, multiple genes may impact the ability of the HRR pathway to repair DSBs. Specifically, *BRCA1/2* alterations are not necessary to cause the HRD phenotype; alterations in other HRR-related genes (i.e., *RAD51C* and *PALB2*) have also been associated with the HRD phenotype [8]. However, the set of necessary and sufficient genetic and epigenetic alterations that drive the clinical manifestation of the HRD phenotype—and thus potential sensitivity to DNA damaging therapies (PARP inhibitors) in specific cancer indications—has yet to be comprehensively determined.

Additional biomarkers, outside of mutations in *BRCA1/2*, are needed to better characterize the HRD phenotype and to identify patients without *BRCA1/2* mutations who are most likely to benefit from DNA repair-targeting therapies. One such biomarker is the presence of a genomic scar which is created when HR-deficient cells are unable to repair DNA damage. Genome-wide loss-of-heterozygosity (gwLOH) measures genomic scarring by calculating the percent of the profiled genome with loss of at least one allele. gwLOH has demonstrated clinical benefit in detecting HRD in ovarian cancers when used either independently [9] or in combination with measures of telomeric allelic imbalance (TAI) and large-scale state transitions (LST) [10]. Many of these DNA-based HRD biomarkers are calculated excluding regions with aneuploidy, defined by loss or gains of chromosomes or chromosome arms, which is considered to be a confounding variable that may inflate gwLOH [9, 11–13].

Additional DNA-based approaches for identifying the HRD phenotype include the assessment of mutational signatures using base-substitutions, indels, or rearrangements [8, 14]. Using whole-genome sequencing data, mutational signatures have suggested 20–30% more breast cancer patients may harbor HRD than what is detectable using the *BRCA1/2* genotype alone [14–16]. While DNA-based HRD biomarkers have demonstrated some preliminary evidence in predicting PARP inhibitor response outside of breast and ovarian cancer [17–19], these methods have only demonstrated clinical utility in ovarian cancer [20, 21]—with significant ongoing research for breast cancer [16, 22–24]—necessitating new approaches for other cancer types. Alternative approaches to detect HRD include measurement of epigenetic silencing of *BRCA1/2* *[*25*–*27*]* and/or epigenetic or genetic loss of other gene members of the HRR pathway [5, 28, 29]. However, these mechanisms may be tissue-specific, requiring additional research between specific alterations and the HRD phenotype. Further, reliance on fresh tissue for whole-genome sequencing and the measurement of multiple molecular modalities can be impractical and costly in real-world clinical practice.

Functional biomarkers of HRD, such as those assessing RAD51 nuclear localization [30], have received increasing attention given the accumulating evidence for the development of resistance to platinum chemotherapy [31] and PARP inhibitors [32], suggesting that HRD is a dynamic phenotype. DNA-based biomarkers of the HRD phenotype have a strong temporal dependency given that a sufficient accumulation of genomic scars is needed for detection. Additionally, genomic markers of instability may have limited reversibility and could represent the molecular history of the tumor rather than the current state of HRR proficiency. In contrast to DNA-based approaches, gene expression has the potential to capture the dynamic state of HRD in a manner that is independent from genomic scarring, particularly in cancer types outside breast and ovarian cancer, where DNA-based approaches have not yet reached the clinic. Transcriptional signatures have shown promise in predicting BRCA status or genomic scars in prostate and pancreatic cancer [33–35]. However, it has yet to be demonstrated that a transcriptome-based model can generalize across solid tumors.

Here, we present an analytical validation of the Tempus HRD platform comprising two assays: HRD-DNA, which measures gwLOH to predict HRD status in breast and ovarian cancers, and HRD-RNA, a logistic regression model trained on whole-exome capture RNA sequencing data that predicts HRD status across all other solid tumors. Both assays were trained to identify biallelic loss of *BRCA1/2*, defining the HRD-positive class using the canonical definition of HRD rather than using a genomic-scar approach. Using data from a large-scale, real-world cohort, we demonstrate the capabilities of these models to accurately detect HRD driven by *BRCA1/2* loss and non-*BRCA1/2* mechanisms.

## Methods

### Sample selection

Prior to sequencing, a hematoxylin and eosin (H&E) stained slide was prepared for formalin-fixed paraffin-embedded (FFPE) tumor specimens and reviewed by a board-certified pathologist to ensure that adequate tissue, tumor content, and sufficient nucleated cells were present to satisfy the minimum tumor content requirement. A minimum tumor content of 20% was required to result in adequate yield at extraction and to proceed with sequencing. Macrodissection was carried out when deemed feasible by a pathologist to increase the tumor content of a specimen. Macrodissection was required if the tumor percentage was less than 40%, and was performed to increase tumor content in some instances.

Sample metadata, specifically tumor purity and cancer cohort labels, was determined by board-certified pathologists. Sample status (i.e.*,* primary, metastatic, lymph node) was determined using a rule set based on heuristics between ICD-10 diagnosis codes and ICD-09 codes for anatomical biopsy site locations. Tissue sites provided by external pathology reports were mapped to ICD-09 codes. Samples that were unambiguously primary samples (e.g., ovarian cancer biopsied from the ovary) were labeled as “Primary”. Samples that were unambiguously metastatic samples (e.g.*,* ovarian cancer biopsied from the liver) were labeled as “Metastatic”. Samples biopsied from a regional lymph node were labeled “Intermediate - Lymph Involvement”. Samples with incomplete biopsy site location information provided on an external pathology report were labeled “Intermediate - Missing Data”.

For orthogonal concordance testing between HRD-DNA and 1p FISH results, samples were ensured to be gliomas biopsied from the brain, with at least 40% tumor purity, and curated with positive or negative FISH results performed on chromosome 1p within 6 months of collection of the biopsy used for xT sequencing.

### DNA and RNA sequencing

A representative sample of de-identified records from 48,843 FFPE tumor samples across 42 solid tumor cancer types with DNA and/or RNA sequencing data were selected from the Tempus Oncology Database. All underwent DNA sequencing based on the Tempus xT targeted panel (*n* = 48,827), Tempus xO (*n* = 9), or Tempus xE whole-exome panel (*n* = 17). When available, a blood sample was used for identifying germline alterations and LOH. For this cohort, 31,019 samples had a matched normal sample available. Of the 48,843 samples with DNA sequencing, 47,997 had RNA sequencing available.

Sample preparation, DNA sequencing, and RNA sequencing for each assay were conducted as previously described [36–40]. For DNA-seq, 50–300 nanograms (ng) of DNA for each tumor sample are sheared to an average size of 200 base pairs (bp), hybridized to the xT probe set, captured using Streptavidin-coated beads and amplified. Amplified target-captured DNA tumor libraries are sequenced to a target depth of 500x on Illumina NovaSeq 6000 before being mapped to Ensembl GRCh37 (Release 75) using Novoalign (Novocraft, Inc.). The xT assay reports on single nucleotide variants, insertions/deletions, and copy number variants across 595–648 genes, which cover 3.6 Mb of genomic space.

The Tempus xT RNA-seq protocol uses exome-capture via IDT xGen probes (> 415,000) spanning > 19,000 genes, with at least 50 ng of extracted RNA required before proceeding to library preparation and a minimum depth of 30 million reads (also sequenced on a NovaSeq 6000). Transcript level abundances are derived from Kallisto (v.0.44.0) [41, 42] pseudoalignments to the Ensembl GRCh37 (Release 75) and normalized for transcript length, GC content, and library size; batch correction is performed for samples sequenced with different probe designs [43]. Gene-level abundances are obtained by summing only transcripts labeled as protein coding by Ensembl. All samples included in these analyses passed QC metrics, including minimum read depth, mapping rate, and duplication rate.

Of the total samples that were assessed, 2058 breast cancer and 1216 ovarian cancer FFPE tumor samples with at least 20% tumor purity underwent tumor-normal matched DNA sequencing with the latest xT version. These breast and ovarian samples were used for training, evaluation, and exploratory analyses for HRD-DNA, and were all run on the latest assay version to ensure consistent probe design and bioinformatics pipelines. Genomic data from the xT assay was analyzed for variants, fusions, rearrangements, copy number, and loss-of-heterozygosity.

### HRD label annotation

Before developing the HRD-DNA and HRD-RNA models, samples were labeled as BRCA*-*biallelic, HRR-wild-type (WT), or HRD-ambiguous based on their mutational status for both *BRCA1/2* and a subset of HRR-related genes. Samples with biallelic loss of *BRCA1* or *BRCA2* were labeled as BRCA-biallelic. Biallelic loss was defined as either (a) homozygous deletion, (b) a pathogenic germline or pathogenic somatic mutation with overlapping LOH of the other allele, or (c) a co-occuring pathogenic germline and pathogenic somatic mutation. HRR-WT samples were defined as samples that had no detected pathogenic mutations, including variants with a low variant allele frequency (VAF), variants of unknown significance (VUS), fusions, copy loss, or LOH in *BRCA1, BRCA2, CDK12, PALB2, RAD51B, RAD51C*, or *RAD51D*.

**Fig. 1:**
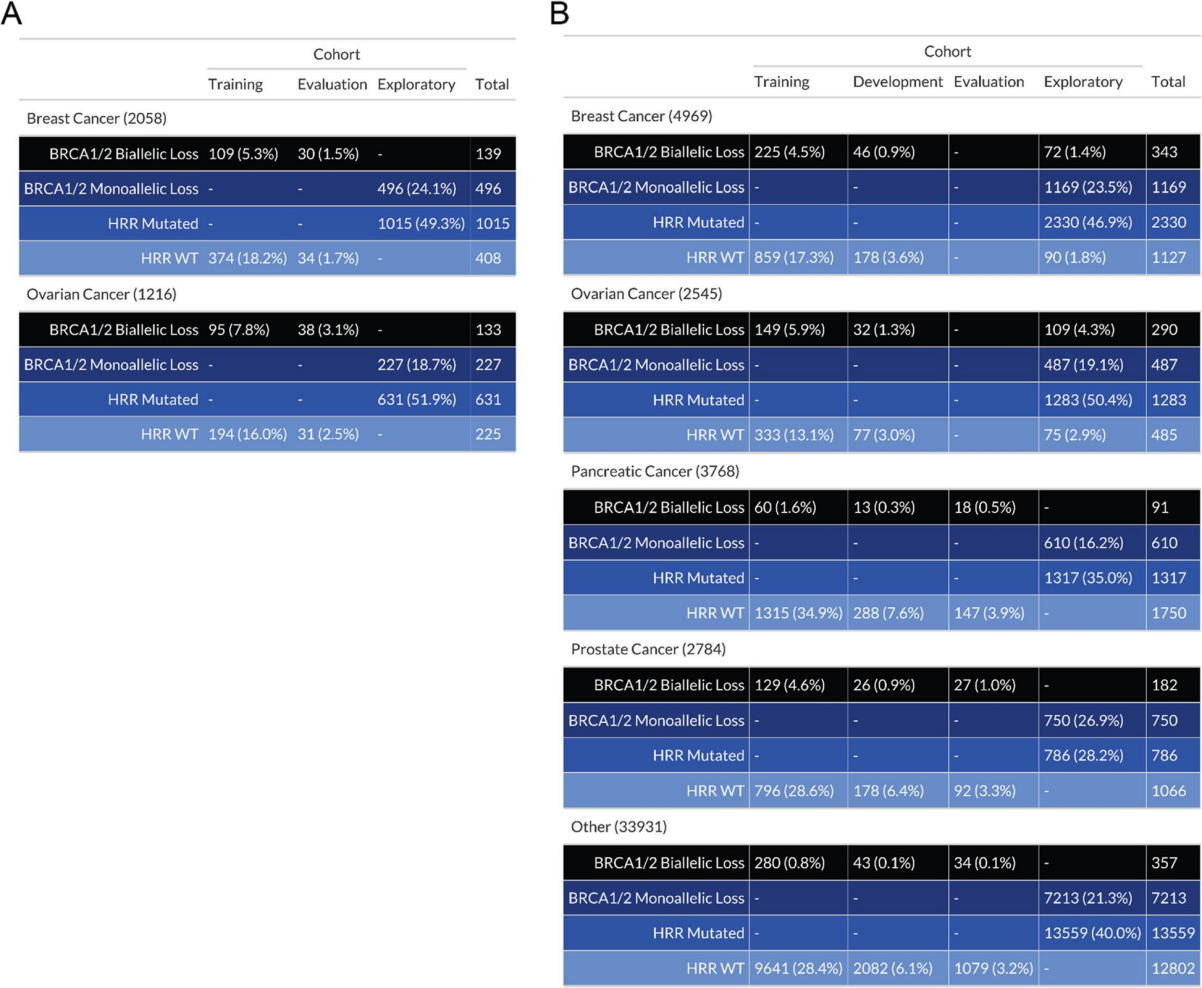
Sample composition for model training, development, evaluation, and exploratory sets by HRR mutation status and cancer type. **A** HRD-DNA. **B** HRD-RNA. To train, develop and evaluate the HRD-DNA and HRD-RNA models, *BRCA1/2* biallelic loss and HRR-WT samples were randomized into the training, development and evaluation sets, while samples with *BRCA1/2* monoallelic loss or HRR alterations (monoallelic or biallelic) in a select number of genes, were assigned to the exploratory set. Samples in the evaluation set were used to test final model performance. Samples in the exploratory set were used to determine overall rates of samples called HRD+ and enrichment of HRR mutations in HRD+ calls. Development sets were only utilized in the HRD-RNA test. Percentages were calculated as a function of the total samples in each cancer cohort for each test

Samples that did not meet the criteria for the BRCA-biallelic or HRR-WT groups were labeled HRD-ambiguous, which fell into two major categories: *BRCA1/2* monoallelic loss or HRR mutated with any alteration in *CDK12, PALB2, RAD51B, RAD51C,* or *RAD51D*. Samples with mutations in these HRR genes were excluded from the HRR-WT group based on their reported associations with HRD status and enrichment for HRD+ calls in initial model iterations [44–50]. Samples from patients treated with PARP inhibitors at any point in their clinical history were also considered HRD-ambiguous, regardless of mutation status, due to the potential for resistance to develop in response to treatment as well as to allow for future model validation with clinical data. Additionally, samples with BRCA reversion mutations (identified by clinical scientists) were also considered HRD-ambiguous. These HRD-ambiguous samples were excluded from model training, development, and evaluation, but were used for exploratory analyses. Overall, ~ 75% of eligible samples were considered HRD-ambiguous (Fig. 1, Supplemental Fig. 1) and therefore excluded from the model training and evaluation sets to ensure the development of a robust model; these samples were, however, included in downstream analyses of model predictions.

### DNA variant, copy number, loss-of-heterozygosity, and fusion annotations

DNA variants, copy number, loss-of-heterozygosity, and fusions were annotated using a combination of bioinformatics pipelines and manual clinical scientist filtering, as described in Beaubier et al. [36]. A minimum 5% VAF was used for calling variants. All BRCA reversion mutations were identified by clinical scientists. Samples that had variants with < 5% VAF (~ 90 samples) in select homologous recombination genes of interest (*BRCA1/2* or *CDK12/PALB2/RAD51B/C/D*) were considered to be HRD-ambiguous. Preliminary analyses found that a number of samples harboring a low VAF pathogenic *BRCA1/2* variant (which would by the 5% VAF cutoff not be called a variant) were predicted as HRD+ while initially being included in the HRR-WT class. To determine homozygous deletions, samples required either (a) evidence of four consecutive probe regions or (b) 20% of the probed length of the gene to have evidence of deletion. A germline or somatic variant with LOH was considered as a biallelic loss when LOH was detected in the same probe region as the detected variant. For fusion events to pass quality control, the fusion was required to present at least five reads of evidence within the DNA-seq data.

### Calculation of gwLOH

The gwLOH calculation required calculation of aneuploid regions followed by the calculation of the total fraction of bases within probe regions with observed LOH. Percent probe loss was calculated as the number of probes with evidence of LOH on a chromosome arm divided by the total number of probes for the chromosome arm. After excluding probe regions on chromosome arms with ≥80% probe loss and probe regions on sex chromosomes, gwLOH was calculated as the total number of sequenced bases in probe regions with LOH divided by total number of bases covered by all probe regions within the assay. Regions with homozygous deletions were considered to have LOH.

### HRD-RNA model preprocessing and training

47,997 samples were eligible for HRD-RNA model development; BRCA-biallelic and HRR-WT samples were stratified by cancer type and HRR mutation status and then randomly assigned at a 12:2:1 ratio to the training, development, and evaluation sets, respectively (Fig. 1B). Normalized gene abundance values for each gene were standardized by removing the mean and scaling to unit variance. These gene expression values were the input to a logistic regression model with L2 regularization and weighting of the positive class. The optimal regularization strength (where the parameter C is the inverse of the regularization strength), positive class weighting (to account for the overrepresentation of WT cases in the training cohort), and number of genes were determined through repeated training and evaluation using the training and development sets. Each repetition used eleven of the twelve training folds to learn the mean and variance scaling parameters and train the model. This preprocessing and modeling pipeline was evaluated on the development set. Each of the twelve folds in the training data was excluded exactly once. After all twelve repetitions, the F1-scores were averaged together to create a single score for a single set of hyperparameter values, which served as the objective function for hyperparameter tuning. 50 sets of hyperparameters were evaluated; five were randomly seeded to ensure sufficient coverage of the hyperparameter space. During hyperparameter tuning, the next set of hyperparameter values to evaluate was selected using Bayesian optimization based on the mean F1-scores of the preceding hyperparameter evaluations [51]. Tuning the HRD-RNA model revealed the optimal set of hyperparameters as an inverse regularization strength of 0.0009 (strong penalty for large parameters), class weight of 11.182 (upweighting of the HRD+ class), and 20,000 genes (Supplemental Fig. 7). These hyperparameter values were used to train the final HRD-RNA model on all twelve training folds. The HRD-RNA model was implemented in python using the *sklearn* logistic regression function with default parameters except as specified above [52].

### Transformation into HRD-RNA scores

The final HRD-RNA score was created by transforming the HRD-RNA logistic regression log-odds values using a logistic function with a maximum value of 100, a logistic growth rate of 1, and a midpoint of 0.72. The midpoint was chosen to optimize the F1-score for distinguishing BRCA-biallelic and HRR-WT samples on the combined training and development sets (Fig. 3A). The final HRD-RNA scores have values from 0 to 100, with a score of less than 50 indicating a prediction of HRD-, and a score of greater than or equal to 50 indicating a prediction of HRD+.

$${HRD}_{RNA}\ Score=\frac{L}{1+{e}^{-k\left(x-{x}_0\right)}}=\frac{100}{1+{e}^{-1\left(x-0.72\right)}}$$

### Statistical analyses

One-way Fisher’s exact tests were used to test for enrichment, i.e. enrichment of BRCA-monoallelic samples with HRD+ calls. To correct multiple hypothesis testing, Bonferroni correction was used to calculate a false-discovery rate, implemented by the *p.adjust()* function from the *stats* package in R [53, 54]. All correlation coefficients and *p-*values represent Pearson correlations implemented by the *cor.test()* function from the *stats* package [54].

## Results

### HRD-labels assigned based on HRR-gene mutation status

A superset of 48,843 tumor samples with targeted DNA-seq and full-transcriptome RNA-seq was used in either model training, development, evaluation, or exploration for the HRD-DNA or HRD-RNA models (Fig. 1). Samples were labeled as either HRD+, HRD-ambiguous, or HRD-, with HRD+ defined as BRCA-biallelic, HRD- as no mutations in *BRCA1, BRCA2, CDK12*, *PALB2*, *RAD51C*, or *RAD51D*, and HRD-ambiguous as BRCA-monoallelic or HRR-mutated (Methods). For reference, samples with any mutation or LOH in *BRCA1, BRCA2, CDK12*, *PALB2*, *RAD51B*, *RAD51C*, or *RAD51D* were considered to be HRD-ambiguous and were not included in the model training, development, or evaluation sets. HRD+ and HRD- labeled samples were randomized into the training, development, and evaluation sample sets. While HRD-ambiguous samples were not used for development or analytical validation, they were used for downstream analyses to demonstrate the utility of the HRD-DNA or HRD-RNA models to identify an HRD signal outside *BRCA1/2* loss.

Among breast cancer samples, 6.8% were annotated as BRCA-biallelic for the HRD-DNA model cohort and 6.8% were BRCA-biallelic for the HRD-RNA model. Additionally, 10.9 and 11.5% of ovarian cancers were annotated as BRCA-biallelic for the HRD-DNA and HRD-RNA model cohorts, respectively (Fig. 1A). These prevalences are slightly lower than what has previously been observed for the frequency of a somatic or germline mutation in breast (7.8%) and ovarian (20%) cancers [55, 56]. This discrepancy is likely attributable to our requirement for biallelic BRCA alterations and to cohort differences found in the Tempus Oncology Database [56, 57]. Our observed prevalence of *BRCA*-deficiency in prostate and pancreatic cancers is 6.5 and 2.4%, which is similar to the values reported by others: 6.2 and 3.4% respectively [58, 59].

For the approximately 75% of samples that were designated as HRD-ambiguous, the majority of samples were excluded from the HRR-WT class due to monoallelic LOH in either *BRCA1/2* or an HRR gene (Supplemental Fig. 1). Samples with LOH were included in the HRD-ambiguous class given the potential for co-occurring alternative mechanisms of BRCA-deficiency (i.e.*,* promoter methylation, a low VAF, or a VUS), limiting the ability to apply a high-confidence HRD label.

### Aneuploidy exclusion and gwLOH calculations for HRD-DNA

The HRD-DNA model was designed to predict the HRD status of breast and ovarian tumor samples using gwLOH, excluding aneuploid chromosome arms. To detect aneuploidy and correct for this potential confounder, we determined the optimal probe loss fraction associated with chromosome arm deletion (and therefore excluded from the gwLOH calculation) and the optimal gwLOH threshold to distinguish BRCA-biallelic (HRD+) samples from HRR-WT (HRD-) samples for both breast and ovarian cancers (Supplemental Fig. 2A). We found that model performance, measured by F1-score (the harmonic mean of precision and recall), was more sensitive to the gwLOH threshold than probe loss threshold, and optimal probe loss thresholds were 78 and 84% for breast cancer and ovarian cancer, respectively. For biological consistency, and given the breast cohort is approximately twice as large as the ovarian cohort, a probe loss threshold of 80% was selected to identify chromosome arms lost due to aneuploidy for both cohorts. The chosen probe loss threshold was validated by applying it to glioma samples that received fluorescence in situ hybridization (FISH) to assess genetic deletion of chromosome 1p (Supplemental Fig. 2B), which is often used to diagnose oligodendrogliomas [60–62]. Samples with negative 1p FISH results had a significantly lower fraction of probes lost compared to 1p FISH positive (*p* < 2.2e-16) (Supplemental Fig. 2C). The chosen probe loss threshold of 80% achieved 89% concordance with 1p FISH results, and samples with > 80% probe loss were significantly enriched for 1p FISH positivity (Fisher’s exact test, *p-*value = 1e-31) (Supplemental Fig. 2D). Note that a lack of 100% concordance with FISH results is to be expected from our methodology given that FISH for 1p deletion usually relies on a single probe that covers only a fraction of the chromosome arm and can therefore result in false positives [63]. Together, these results demonstrate that the probe loss method accurately identifies chromosome arms lost due to aneuploidy.

**Fig. 2:**
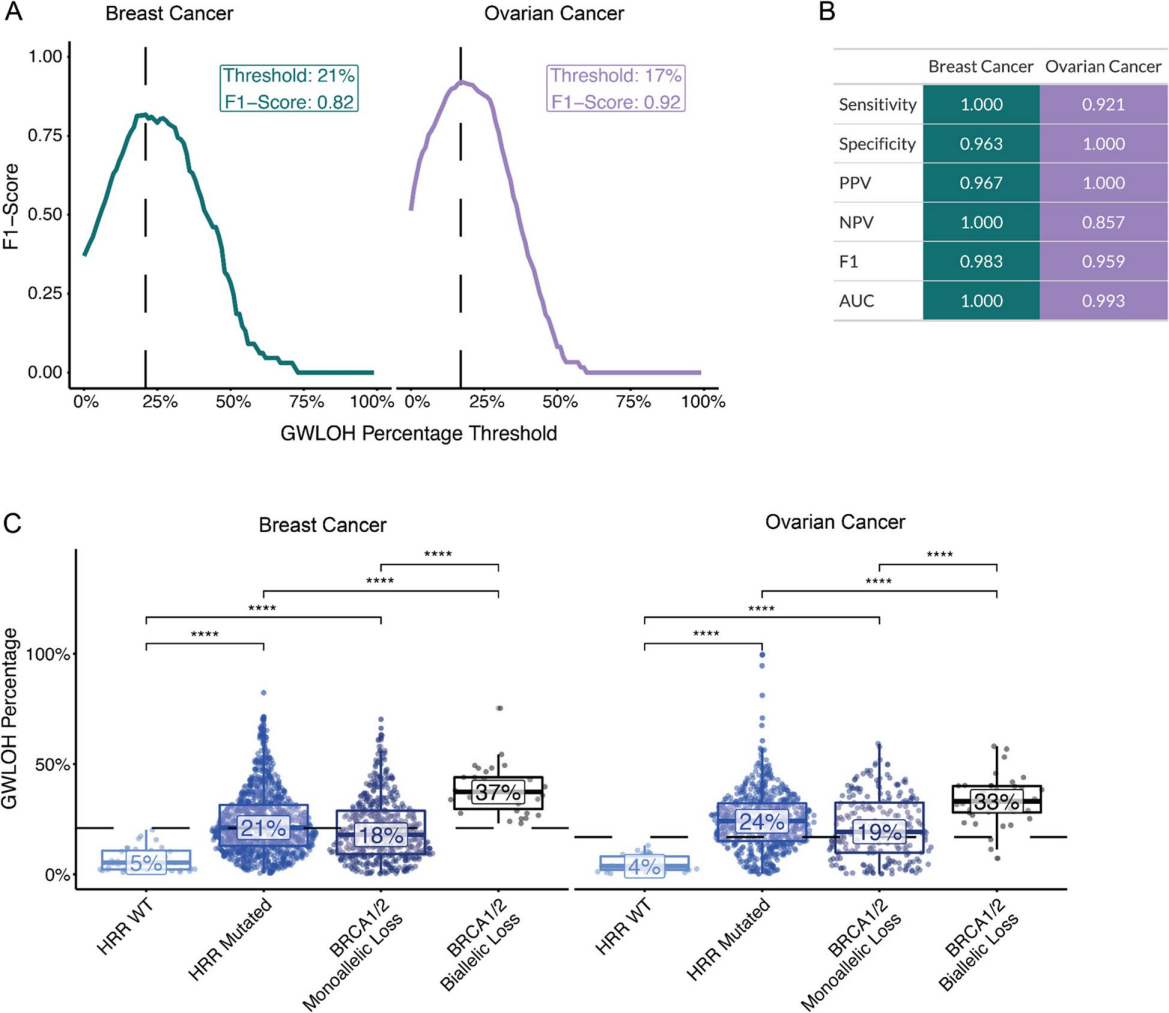
The HRD-DNA model predicts HRD status from gwLOH. **A** Thresholds for calling a sample HRD+ for breast and ovarian cancer were set based on the maximum F1-score within samples in the training set. **B** Metrics used to assess HRD-DNA performance within the evaluation set. The confidence interval for breast cancer was [1, 1] and ovarian cancer AUC was [0.979, 1]. **C** Distribution of gwLOH scores across different HRR genotypes in the evaluation set. HRR mutated samples contain samples with either monoallelic or biallelic loss in a select number of HRR genes. Values in the box represent the median gwLOH percentage within each HRR genotype (**** *p-*value < 0.0001 for Wilcoxon test). Dotted lines are the thresholds chosen in (A) for each cancer type. Statistical differences between HRR-WT and BRCA biallelic loss were not shown, but all were significant (*p-*value < 0.0001 for Wilcoxon test)

Finally, the training samples were used to identify the optimal gwLOH score—excluding aneuploid chromosome arms—for calling a sample HRD+ or HRD-. The gwLOH threshold was determined as the threshold that best distinguished the BRCA-biallelic from HRR-WT samples, measured by F1-score. The optimal gwLOH threshold was 21 and 17% for breast and ovarian cancer, respectively (Fig. 2A). The evaluation set of samples were then used to evaluate the performance of the chosen probe loss and gwLOH thresholds, yielding robust performance metrics: sensitivity (breast = 1, ovarian = 0.921), specificity (breast = 0.963, ovarian = 1.0), positive predictive value (PPV) (breast = 0.967, ovarian = 1.0), negative predictive value (NPV) (breast = 1.0, ovarian = 0.857), F1-score (F1: breast = 0.983, ovarian = 0.959), and area under the receiver-operating characteristic curve (AUC) (breast = 1.0, ovarian = 0.993) (Fig. 2B). The HRD-DNA model had a lower sensitivity and NPV for ovarian cancer relative to breast cancer in the evaluation set, suggesting there may be a greater fraction of patients with low gwLOH that are HRD+ in ovarian cancer (Fig. 2C). Finally, HRR-WT samples had significantly lower gwLOH compared to HRD-ambiguous samples in both HRR-mutated (Wilcoxon test; *p-*value_breast_ = 2e-14, *p-*value_ovarian_ = 4e-16) and *BRCA1/2* monoallelic samples (Wilcoxon test; *p-*value_breast_ = 1e-10, *p-*value_ovarian_ = 4e-11). Samples were predicted HRD+ at a lower rate for HRR-WT samples (breast = 2.9%, ovarian = 0%) compared to HRR-mutated (breast = 56.7%, ovarian = 69.3%) and *BRCA1/2* monoallelic (breast = 48.8%, ovarian = 56.8%) — suggesting other potential drivers of the HRD-phenotype (Supplemental Fig. 3A). The gwLOH biomarker separated samples in the evaluation set with *BRCA1/2* biallelic loss from samples with no evidence of mutations in *BRCA1/2* or a subset of HRR genes with 100% sensitivity and 96.3% specificity in breast and with 92.1% sensitivity and 100% specificity in ovarian cancer (Fig. 2B).

### HRD-RNA model training and evaluation

**Fig. 3:**
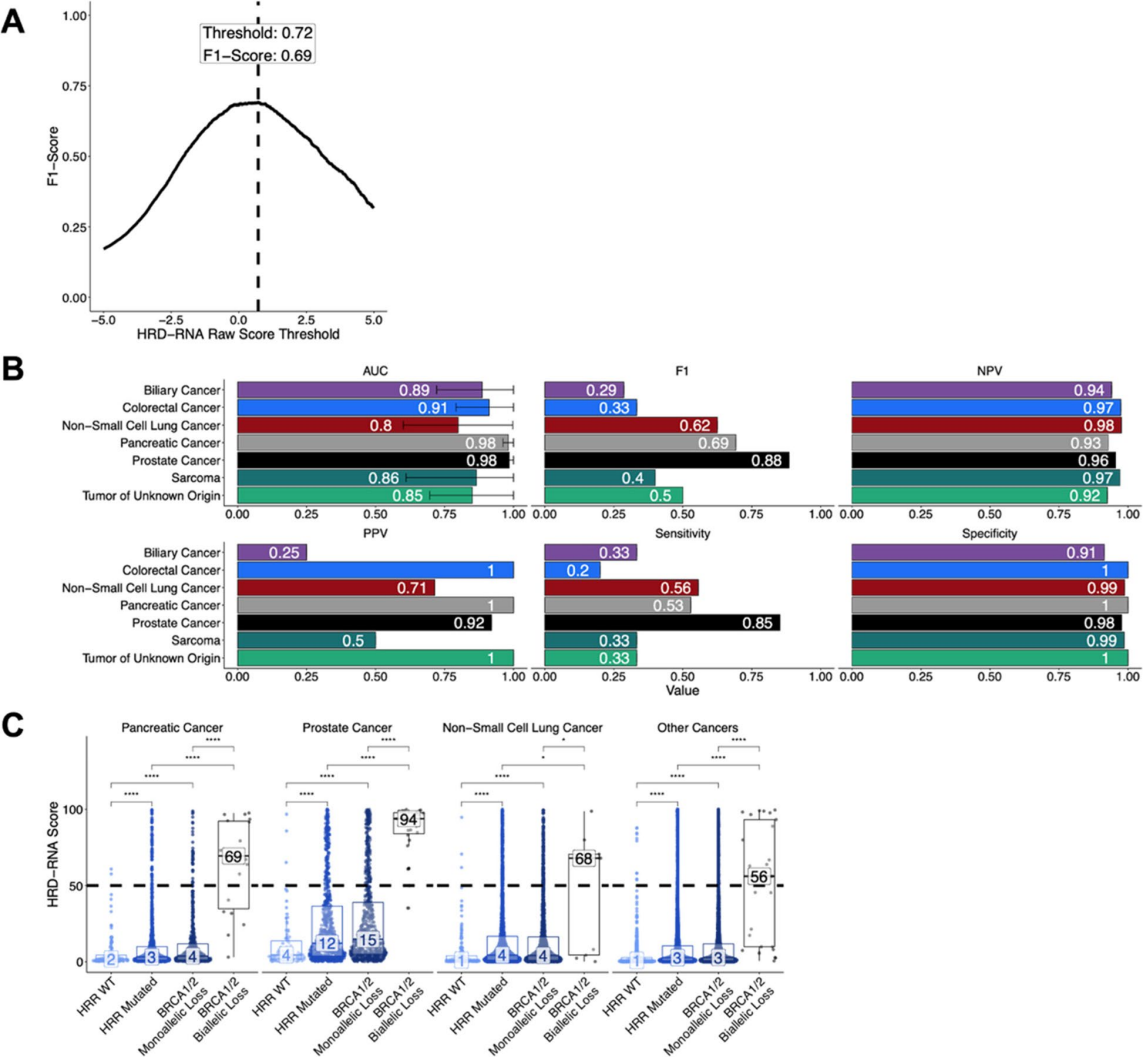
The HRD-RNA model determines HRD-status for cancer cohorts outside breast and ovarian cancer using a logistic regression model trained on RNA-seq data. **A** The threshold for calling a sample HRD+ was set as the raw RNA score that had the maximum F1 score on the training and development samples. For final reporting, the raw score was transformed to the final HRD-RNA, where a score of 50 represents the chosen threshold (Methods). **B** Metrics used to assess HRD-RNA model performance across cancer cohorts for samples within the evaluation set. **C** Distribution of HRD-RNA scores across different HRR genotypes in the evaluation set for cancer indications with > 3 BRCA-deficient samples in the evaluation set. Dotted line represents the threshold chosen in (A). Values in the box represent the median HRD-RNA score. Differences determined by Wilcoxon test (* *p-*value < 0.05, **** *p-*value < 0.0001). Statistical differences between HRR-WT and BRCA biallelic loss were not shown. Statistical differences between HRR-WT and BRCA biallelic loss were not shown, but all were significant (*p-*value < 0.0001 for Wilcoxon test). AUC: Area Under the Curve. NPV: Negative Predictive Value

While DNA-based approaches for detecting HRD have demonstrated utility in the clinic for ovarian cancer [20, 21] and are currently under investigation for breast cancer [16, 22–24], there is a need for a pan-cancer biomarker of HRD that can generalize to other tumor types. Here, we utilize RNA-sequencing data with a logistic regression model to identify a pan-tumor gene expression signature of HRD, here called the HRD-RNA model. The same sample labels that were assigned for the HRD-DNA model were also used for the HRD-RNA model, relying on biallelic loss of *BRCA1/2* to define the HRD-positive class for model training. BRCA-biallelic and HRR-WT samples from all cancer types were included in the training and development sets for the HRD-RNA model, including breast and ovarian cancers (Fig. 1B). The HRD-RNA model was evaluated for cancer types that included at least 3 BRCA-biallelic samples in the evaluation set (Fig. 3B). Across these cancer types, the model achieved a PPV of 25%, indicating that only a fraction of the patients predicted HRD+ exhibited *BRCA1/2*-deficiency. The highest AUCs on the evaluation set were in prostate (0.98) and pancreatic (0.98) cancer, which is unsurprising given that tumor pathogenesis in these cohorts has been previously associated with BRCA status [64, 65]. For prostate and pancreatic cancers in the evaluation and exploratory sets, there was strong separation between the BRCA-biallelic and HRR-WT samples (Wilcoxon test; *p-*value_Prostate_ = 1e-14, *p-*value_Pancreatic_ = 6e-11), and between the HRR-WT and HRD-ambiguous samples (Wilcoxon test; *p-*value_Prostate_ = 4e-9, *p-*value_Pancreatic_ = 6e-8) (Fig. 3C).

While other cancer cohorts — biliary, colorectal, non-small cell lung cancer, sarcoma, and cancers of unknown primary (tumors of unknown origin) — had a lower prevalence of *BRCA1/2* alterations, previous work has suggested that these cohorts may exhibit the HRD phenotype and respond to PARP inhibitors (Fig. 1B) [66–68]. We hypothesized that tumors from patients with these cancer types may exhibit the HRD phenotype in the absence of BRCA loss. For these cohorts, we observed lower overall performance relative to BRCA status when compared to pancreatic and prostate cancer (Fig. 3B).

Across all cohorts evaluated using the HRD-RNA model, the number of samples with BRCA-biallelic loss in the training and development sets was positively correlated with the F1-score (R = 0.92, *p-*value = 3e-3) and sensitivity (R = 0.95, *p-*value = 1e-3) of the model on the evaluation set (Supplemental Fig. 4). This result suggests a biological and/or modeling constraint in cancer cohorts with few BRCA-biallelic samples — either BRCA deficiency may be a poor surrogate for HRD status or there may be insufficient BRCA-biallelic samples to identify a signal. The high fraction of samples that were HRD-ambiguous and predicted HRD+, i.e. 13.1% of BRCA-monoallelic and 11.7% of HRR-mutated prostate cancers, suggests that mutations in other HRR pathway genes or epigenetic modifications (i.e.*,* hypermethylation) may drive the HRD-phenotype in cancer types not traditionally associated with BRCA-status (Supplemental Fig. 3; Fig. 3C).

### The HRD-DNA and HRD-RNA models capture an underlying HRD-phenotype consistent with the literature

Both the HRD-DNA model and the HRD-RNA model captured biallelic loss of *BRCA1/2* with high sensitivity and specificity in BRCA-associated tumors (breast, ovarian, pancreatic, and prostate cancer) (Fig. 2B; Fig. 3B). We hypothesized that some fraction of the ~ 75% of samples annotated as HRD-ambiguous would be predicted HRD+ (Fig. 1; Supplemental Fig. 1); these HRD-ambiguous samples had higher HRD scores (Fig. 2C, Fig. 3C) and a higher frequency of HRD+ predictions compared to HRR-WT samples (Supplemental Fig. 3) by both the HRD-DNA and HRD-RNA models. Together, these findings further suggest that there is an underlying HRD-phenotype not captured by BRCA biallelic loss alone.

**Fig. 4:**
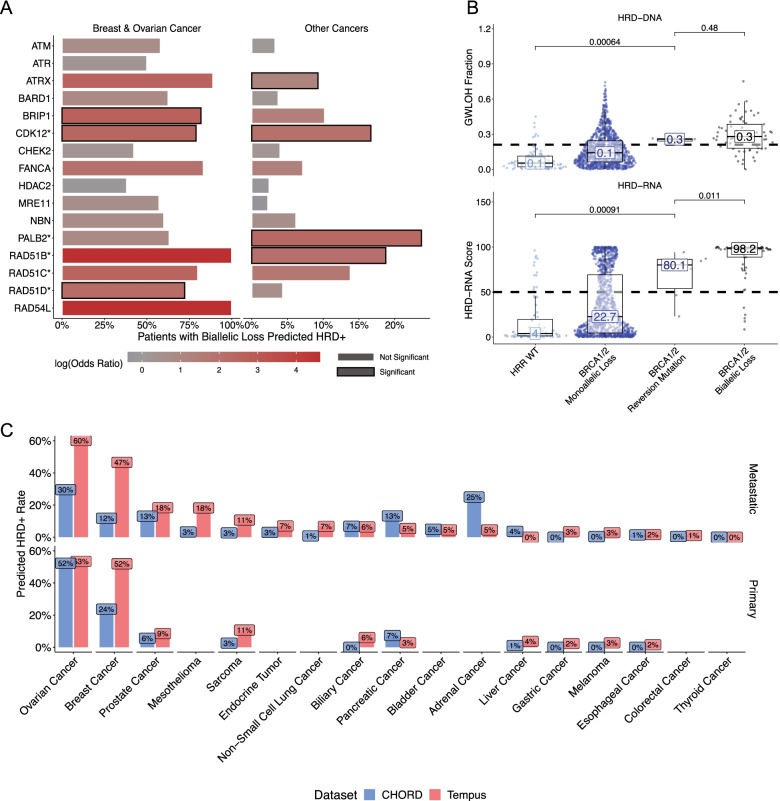
The HRD-DNA and HRD-RNA models are enriched for a HRD-phenotype and are concordant with published HRD+ rates. **A** Enrichment for HRD+ calls in samples with biallelic loss of HRR genes for breast cancer and ovarian cancer (HRD-DNA), and other cancers (HRD-RNA). Enrichment was calculated using a Fisher’s exact test comparing samples from the HRD-ambiguous and HR-WT samples that has biallelic loss of specific HRR gene versus all other samples. Significance was determined as *p-*value < 0.05. **B** Distribution of GWLOH percentage (top) and HRD-RNA scores (bottom) of breast cancer samples with *BRCA1/2* reversion mutations. Significance shown for two-sided Wilcoxon-test. **C** Predicted rates of HRD+ samples across cancer types compared to published rates (CHORD), stratified by primary and metastatic samples. For breast and ovarian cancer, the HRD-DNA model was used to determine rates of HRD+ samples. For all other cancer cohorts, the HRD-RNA model was used

Outside *BRCA1/2* alterations, samples predicted HRD+ by each HRD model were enriched for biallelic loss of HRR-related genes, which would suggest an alternative mechanism for the HRD-phenotype (Fig. 4A). Here, we defined HRR-related genes more broadly to include: *ATM*, *ATR*, *ATRX*, *BARD1*, *BRIP1*, *CDK12*, *CHEK2*, *FANCA*, *HDAC2*, *MRE11*, *NBN*, *PALB2*, *RAD51C*, *RAD51B*, *RAD51D*, and *RAD54L*. Significant enrichment of HRD-DNA+ predictions was observed in samples with biallelic loss in *BRIP1* (False-discovery rate corrected *p-*value [FDR] = 4.8e-7), *CDK12* (FDR = 3.1e-5), and *RAD51D* (FDR = 2.7e-4). Previous studies have also demonstrated that biallelic loss in *BRIP1* or *RAD51D* is associated with a higher gwLOH score in BRCA-associated cancers [9]. In other cancer types, there was a lower overall fraction of samples predicted HRD+ across these HRR genes, highlighting the overall lower frequency of HRD in these cancer types (Fig. 4A). However, there was significant enrichment for HRD-RNA+ predictions in samples with biallelic loss of *ATRX* (FDR = 9e-8), *CDK12* (FDR = 4e-5), *FANCA* (FDR = 2e-2), *PALB2* (FDR = 3e-8), and *RAD51B* (FDR = 5e-4). Enrichment of HRD+ calls in samples with biallelic loss of HRR genes highlights the utility of both the HRD-DNA and HRD-RNA models in identifying a number of potential drivers of the HRD phenotype that are independent of *BRCA*1/2 biallelic loss.

The HRD-RNA model, in contrast to the HRD-DNA model, has the potential to capture a dynamic HRD phenotype. For example, one mechanism for PARP inhibitor resistance is via a *BRCA1/2* reversion mutation [69–71]. Though rare, samples with these mutations can serve as a test of the dynamic nature of the HRD-RNA model compared to the HRD-DNA model. In the presented data, breast cancer samples most frequently possessed BRCA reversion mutations (*n* = 7); these cases were excluded from the HRD-RNA model training and development sets. The HRD-RNA model predicted lower HRD scores for samples with a *BRCA1/2* reversion mutation compared to samples with biallelic loss of *BRCA1/2* (Wilcoxon test; *p-*value = 0.002), indicating the ability of the HRD-RNA model to capture a dynamic HRD phenotype (Fig. 4B). On the other hand, there was no significant difference in the HRD-DNA score between samples with a *BRCA1/2* reversion mutation and BRCA-biallelic samples. Although five out of the seven samples with BRCA reversions were predicted to be HRD+ by HRD-RNA, this may be attributable to clonal BRCA reversion (mean VAF: 13.3%), resulting in clonal HRD.

Finally, given the different approaches for predicting HRD status (HRD-DNA and HRD-RNA) and the low PPV for non-BRCA associated cancer cohorts, we compared rates of HRD+ calls between the presented models and the literature (Fig. 3B, Fig. 4B). The Classifier of HOmologous Recombination Deficiency (CHORD) method utilizes genomic features from whole-genome sequencing data with a random forest classifier to determine HRD-status [8]. HRD prevalence across tumor types estimated by CHORD was used as a benchmark for positivity rates predicted by HRD-DNA (breast and ovarian) and HRD-RNA (all other cohorts), stratified by primary and metastatic status. For both primary and metastatic cancers, there was a strong, positive correlation (HRD-DNA: R^2^ = 0.63, *p-*value = 4e-4; HRD-RNA: R^2^ = 0.83, *p-*value = 1e-7) between the predicted frequency of HRD+ samples in the presented models and the respective predicted frequency in CHORD (Fig. 4C; Supplemental Fig. 5). This suggests predicted HRD+ prevalence within each cancer type is supported by other studies.

There were a few notable deviations in predicted HRD prevalence across tumor types. First, the HRD-DNA model reported an HRD+ prevalence of 52 and 61% in primary breast and ovarian cancer, while CHORD presented 54 and 30%, respectively. This difference may be due to the fact that patients sequenced by Tempus are often later-stage and have received more lines of therapy. Moreover, other groups have reported ovarian cancer HRD+ prevalence in the 40–50% range [72, 73]. In metastatic breast cancer, CHORD reported an HRD+ prevalence of 12%, while the HRD-DNA model predicted 46%. Upon further inspection of the cohort used by CHORD, 13.5% of all metastatic breast cancer samples were triple-negative (TNBC), the breast subtype for which HRD positivity is highest [74]. By contrast, the Tempus cohort is enriched for TNBC samples given that these patients have few treatment options and worse outcomes, and are thus more likely to undergo Tempus NGS testing. Indeed, nearly 24% of Tempus breast cancers are TNBC, and, as a result, models predicting HRD status using Tempus data should be expected to report a higher HRD+ prevalence compared to CHORD.

### HRD-DNA and HRD-RNA are robust to confounders

The limit of detection (LOD) for both the HRD-DNA and HRD-RNA models was determined by calculating the sensitivity of both models across different tumor purities. The LOD was set as the lowest tumor purity at which the PPV was greater than 70%. For the HRD-DNA model, PPV was calculated using all breast or ovarian samples that had either *BRCA1/2* biallelic loss or were BRCA-WT. A tumor purity threshold was set at 40% based on the PPV threshold (Supplemental Fig. 6). For the HRD-RNA model, PPV was calculated using all samples, including breast and ovarian samples, that had either *BRCA1/2* biallelic loss or were BRCA-WT and were in the evaluation set. A tumor purity threshold was set at 30% based on the PPV threshold (Supplemental Fig. 6). These data indicate that the HRD-DNA and HRD-RNA models are performant among samples with at least 40 and 30% tumor purity, respectively.

To test whether model performance is tissue-site dependent, the evaluation set was stratified by sample status - primary, metastatic, lymph node, or unknown (Supplemental Table 1). For both HRD-DNA and HRD-RNA, model performance was similar in each sample stratification. For HRD-DNA, F1-score ranged from 0.933–1.0 and sensitivity ranged from 0.875–1.0. For HRD-RNA, F1-score is higher in primary (0.741) compared to metastatic (0.591) samples, mostly driven by higher sensitivity in primary samples (0.690) compared to metastatic (0.448) samples. However, AUC was similar between primary (0.956) and metastatic (0.953) samples, compensated for the higher specificity in metastatic (0.994) samples over primary (0.990) samples. Overall, HRD-DNA and HRD-RNA performance is robust to biopsy site.

**Fig. 5:**
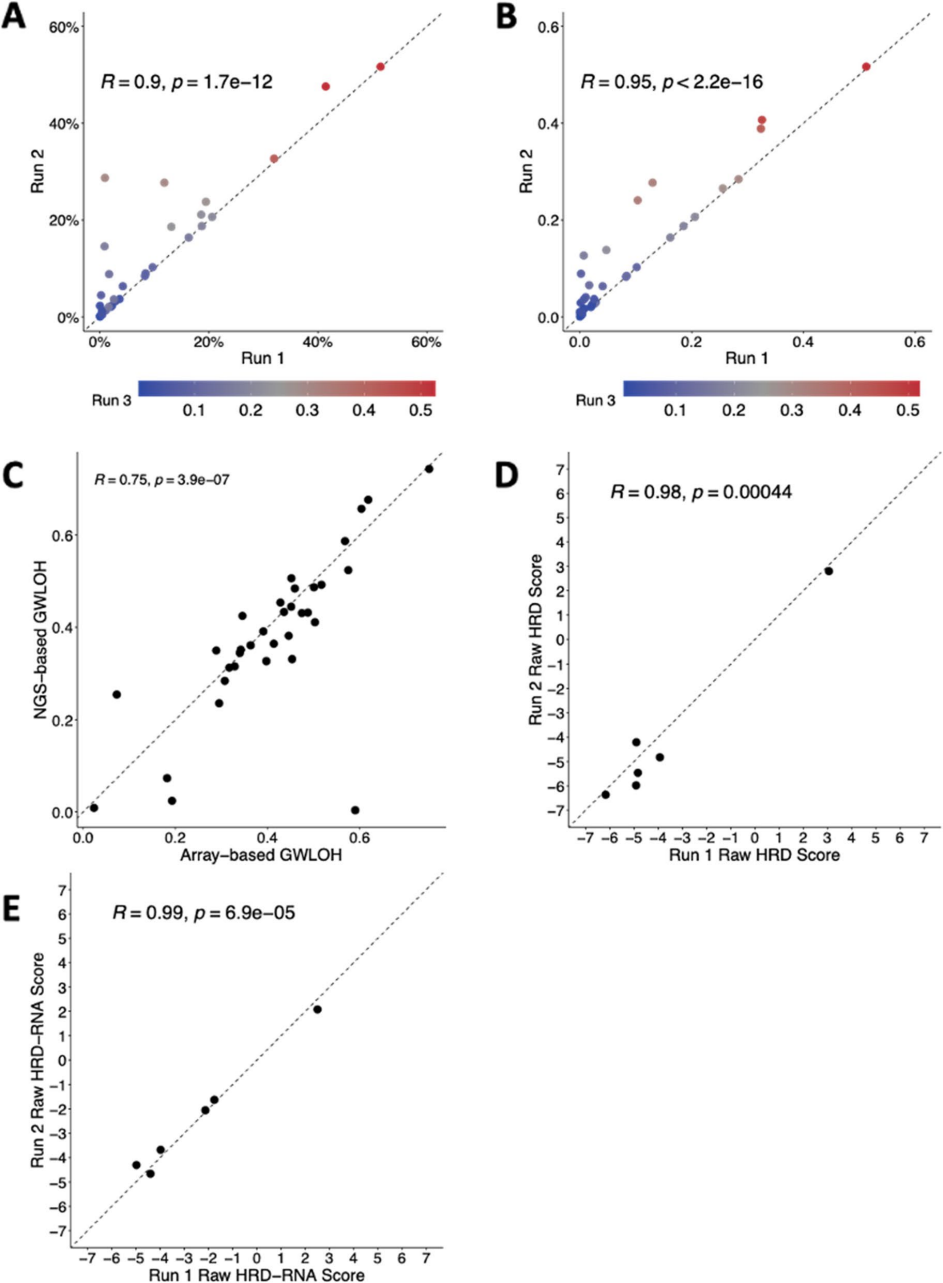
Inter- and intra-assay concordance for the HRD-DNA and HRD-RNA models. **A** Intra-assay concordance of HRD-DNA for 32 samples each run in triplicate. **B** Inter-assay concordance of HRD-DNA for 34 samples each run in triplicate. **C** Correlation between HRD-DNA and Omni 2.5 BeadChip array based genome-wide LOH. (**D**) Intra assay concordance of HRD-RNA for 6 samples each run in duplicate. **E** Inter assay concordance of HRD-RNA for 6 samples each run in duplicate. Dotted lines are identity lines

Finally, to establish reproducibility across sequencing runs, experiments were run to demonstrate inter- and intra-assay concordance for the HRD-DNA and HRD-RNA models. To demonstrate intra-assay concordance for the HRD-DNA model, 32 samples were sequenced in triplicate in the same DNA sequencing run using the same reagent lot with different barcodes (Fig. 5A). There was a significant correlation for all run comparisons (0.87 < R < 0.97 across all comparisons, *p-*value <1e-11), demonstrating intra-assay concordance. To demonstrate inter-assay concordance for HRD-DNA, 34 samples were sequenced in triplicate on different days, using different instruments, different lab technicians, and at least two manufacturing reagent lots (Fig. 5B). There was a significant correlation for all run comparisons (0.89 < R < 0.98 across all comparisons, *p-*value <1e-12), demonstrating high inter-assay concordance. To orthogonally validate HRD-DNA gwLOH calls, 34 samples were run via Omni2.5 BeadChip copy number array at an external lab [75]. The HRD-DNA score was calculated from both sets of copy calls and was highly correlated (R = 0.75, *p-*value = 3.9e-7; Fig. 5C).

To demonstrate intra-assay concordance for the HRD-RNA model, 6 samples were sequenced in duplicate in the same RNA sequencing run using the same reagent lot with different barcodes (Fig. 5D). There was a significant correlation of the raw RNA output (R = 0.98, *p-*value = 4.4e-4), demonstrating intra-assay concordance. To demonstrate inter-assay concordance for the HRD-RNA model, 6 samples were sequenced in duplicate on different days, using different instruments, different lab technicians, and at least two manufacturing reagent lots (Fig. 5E). There was a highly significant correlation of the raw RNA output (R = 0.99, *p-*value = 6.9e-5), demonstrating inter-assay concordance.

## Discussion

Here, we present two models, HRD-DNA and HRD-RNA, that predict the HRD status of clinical FFPE samples. The models were trained and evaluated on their ability to predict *BRCA1/2*-biallelic from HRR-WT samples, relying on the canonical definition of HRD to define HRD-positive samples for training, rather than other genomic-scar based approaches. Samples that were predicted as HRD+ were also enriched for biallelic loss of other HRR genes and predicted frequencies of HRD+ samples across cancer cohorts are largely in agreement with what has been reported in the literature. The models’ ability to detect an HRD phenotype rather than solely BRCA-loss is critical outside of the so-called non-BRCA associated tumors (breast, ovarian, pancreatic and prostate). Such HRD detection tools also present an opportunity to identify alternative drivers of the HRD phenotype, and potential indications beyond those reported in the literature. HRD biomarkers based on genomic scarring have only thus far demonstrated clinical utility in ovarian cancer [20, 21], while an RNA-based biomarker may be able to capture a gene expression signature that generalizes across tumor types. For example, we found high rates of HRD in sarcomas and mesotheliomas, highlighting that RNA-based biomarkers may provide advantages for capturing a shared HRD phenotype in these histologies. Together, these models provide new biomarkers for HRD-status in solid tumor samples.

The basis for the HRD-DNA model, gwLOH, is known to have different manifestations across tumor types, necessitating cancer-specific thresholds for calling a sample HRD+. For breast and ovarian cancers, there were sufficient BRCA-biallelic samples to set a threshold for HRD-DNA to predict HRD-status. However, other cohorts either had too few BRCA*-*biallelic samples or little difference in gwLOH between BRCA-biallelic and HRR-WT samples, necessitating an alternative approach. For solid tumor indications outside of breast and ovarian, the HRD-RNA model predicts HRD-status using a logistic regression model trained on bulk RNA-seq data from solid tumor FFPE samples. Breast and ovarian cancer had the highest prevalence of BRCA-biallelic samples (Fig. 1); despite these being the dominant cancer cohorts for HRD+ samples, we assumed that the transcriptional signature of *BRCA1/2* mutations and HRD would be consistent across tumor types for predictive power of HRD independently of tumor type. The positive relationship between the number of BRCA-biallelic samples within a tumor type and the model F1-score highlights that model performance, as benchmarked by BRCA mutation status, is superior in cohorts with higher prevalence of BRCA alterations. Cancer types with lower prevalence of BRCA-biallelic samples and a low PPV suggest that biallelic BRCA-loss may not be driving tumorigenesis. While p53 loss has been observed to confound gwLOH measurements [9], there is little understanding of confounding factors for RNA-based HRD approaches. Future work should focus on uncovering alternative causes of HRD, and disambiguating the differences in performance across cancer cohorts with the potential co-occurence of other tumor drivers where biallelic loss of BRCA may be a passenger rather than driver mutation.

The HRD-DNA and HRD-RNA models not only capture BRCA-biallelic samples but also demonstrate enrichment for samples labeled as HRD-ambiguous (Fig. 2C, Fig. 3C) with biallelic loss of other HRR genes (Fig. 4A). While *ATM* was the most commonly lost HRR gene in ovarian, breast, and other cancers, and occurred at a rate similar to *BRCA1/2*- deficiency, HRD+ calls were enriched only in the HRD-DNA model, suggesting that *ATM* loss may be uniquely associated with a high gwLOH phenotype in breast and ovarian cancer but not HRD in other cancer types. In metastatic castration-resistant prostate cancer, clinical trials have shown little to no clinical benefit of PARP inhibitors in *ATM*-mutated patients over standard of care treatment [5, 76]. Other mutations were uniquely enriched in HRD+ predicted cases of breast and ovarian cancers (*BARD1*, *BRIP1*, *MRE11*, *RAD51D*) and cancer cohorts outside breast and ovarian cancer (*RAD51B*), suggesting additional unique drivers of HRD that may be cancer-cohort specific. Monoallelic copy loss of HRR genes was highly prevalent and could not be included in the HRD-ambiguous category without compromising the statistical power of the HRR-WT cohort. Future work should explore the association between specific alterations (i.e.*,* single nucleotide polymorphisms, LOH, deletions), pathogenicity (VUS), contexts (i.e.*,* germline, somatic), and genes (i.e.*, BRCA1/2*, HRR genes) with HRD calls to better understand mechanisms driving the HRD+ predictions.

In both the HRD-DNA and HRD-RNA models, samples with biallelic loss of *CDK12* were enriched for HRD+ predictions across tumor types. Mutations in *CDK12* have been shown to confer sensitivity to PARP inhibitors in breast and ovarian cell lines [44] and clinical trials in prostate cancer [5]. Further, the HRD-RNA model enriched for samples with biallelic loss of *ATRX*, *PALB2, and RAD51B. PALB2* has been recognized to play a role in HRR through interactions with *BRCA1/2* [48] and, more recently, *PALB2* has been implicated as another potential genomic biomarker for HRD [47, 77, 78]. Biallelic loss of *ATRX* has been associated with increased gwLOH in breast but not ovarian or other cancer cohorts [9]. *ATRX* mutations have been shown to inhibit homologous recombination repair in cell lines [79–81], are linked to PARP inhibitor sensitivity in patient-derived xenografts [82], are associated with higher *PARP1* expression in clinical glioblastoma tumors [83], and have shown sensitivity to DNA-damaging treatment in pediatric high-grade glioma patients [84]. Overall, these pan-cancer models of HRD highlight the ability to accurately capture the HRD phenotype and thus generate new hypotheses for other potential genetic drivers of HRD.

Notably, the HRD-RNA model predicted samples with BRCA-reversions to have a significantly lower HRD-RNA score than BRCA-biallelic samples, which was not true of HRD-DNA. This result suggests an RNA-based measure of HRD may capture dynamic changes in HRD phenotype upon tumor evolution. While the HRD-RNA score is significantly lower in this population, the majority of samples would still be predicted HRD+ by the RNA model, highlighting the need for additional data to confirm these findings. Given that PARP inhibitor resistance can be caused by a number of mechanisms that cannot be detected by DNA alone [85], future work should explore the utility of RNA-based approaches for both identifying HRD samples and tracking the emergence of resistance.

Whole-genome sequencing (WGS) is challenging and expensive to implement in real-world clinical practice, but WGS-based models have emerged as a potentially comprehensive tool to capture HRD. Numerous methods based on mutational signatures from WGS have been published in the literature [8, 14]. While future work should explore comparing these methods to the presented HRD-DNA and HRD-RNA methods on a publicly available dataset, such as TCGA, here we compare the prevalence of HRD on a cohort level between Tempus and one such mutational signature on another database. We demonstrated a strong correlation between the predicted frequency of one such WGS-based method, CHORD, and HRD+ frequencies from the presented models in both primary and metastatic samples. Deviations (i.e.*,* ovarian and breast cancer) can be partially explained by differences in HRD rates among different subtypes and enrichment of subtypes with higher rates of HRD+ samples in the Tempus Oncology Database. One notable deviation from the predicted rates of HRD+ samples is in pancreatic cancer where, for both metastatic and primary samples, the predicted rates for HRD-RNA are lower than CHORD. Reported rates of HRD in pancreatic cancer vary between 3 and 30% [19, 35], where RNA-based approaches have been shown to be prognostic and identify other genetic drivers of HRD [35]. Given clinical trials have demonstrated no survival benefit using olaparib to treat pancreatic cancer patients with germline BRCA alterations, there remains a poor understanding of HRD manifestation in pancreatic cancer [86].

Significantly higher rates of HRD+ in mesothelioma (metastatic) and sarcoma (primary and metastatic) were seen with the presented models compared to CHORD. PARP inhibitors are currently being explored in combination with immune checkpoint inhibitors in mesothelioma [87], where improved response was observed in patients with germline mutations in HRR genes [88]. However, little work has been done exploring the role of HRD as a biomarker in sarcomas. A recent study demonstrated improved response to PARP inhibitors in patient-derived soft-tissue xenografts with high *PARP1* expression [89]. Collectively, these observations suggest that HRD-RNA captures a unique but shared HRD signature across cancer types, which may help in clinical translation. Both mesothelioma and sarcoma present new cohorts to explore HRD as a potential biomarker, but more data is needed to determine the role of HRD and its value as a biomarker in these indications.

## Conclusions

The HRD-DNA and HRD-RNA models developed and validated here accurately differentiate BRCA-biallelic from HRR-WT samples and enrich for other genomic events in the HRR pathway. Our findings suggest that while biallelic loss of *BRCA1/2* may be sufficient to detect the majority of cases of the HRD-phenotype in breast and ovarian cancer, more work is needed to identify the relationship between genotype and HRD in other cohorts. The HRD-RNA model sheds light on this relationship by determining a pan-cancer signature of HRD that can be applied to cancer cohorts across numerous and varied subtypes. This may be most valuable as a precision medicine tool in tumor indications with a lower frequency of biallelic BRCA loss. Further work is warranted to determine potential HRD driver events and clinical implications of HRD status across cancer indications. Importantly, future prospective clinical studies are required to assess the clinical utility of the presented HRD biomarkers for predicting response to DNA-damage targeting therapies.

## Supplementary Information

**Additional file 1.**

## Data Availability

Raw data for this study were generated at Tempus Labs. Derived data supporting the findings of this study are available either within the paper or its Supplementary Figures.

## Abbreviations

HRD: Homologous Recombination Deficiency
HRR: Homologous Recombination Repair
DSB: Double-Strand Break
PARP: Poly-ADP ribose polymerase
gwLOH: Genome-wide loss-of-heterozygosity
WT: Wild-type
H&E: Hematoxylin and Eosin
FFPE: Formalin-Fixed Paraffin-Embedded
VAF: Variant Allele Frequency
VUS: Variant(s) of Unknown Significance
FISH: Fluorescence in situ hybridization
PPV: Positive Predictive Value
NPV: Negative Predictive Value
FDR: False-discovery rate (corrected *p-*value)
AUC: Area under the receiver-operating characteristic curve
CHORD: The Classifier of HOmologous Recombination Deficiency
TNBC: Triple-negative breast cancer
LOD: Limit of detection
WGS: Whole-genome sequencing
